# 28 day post-operative persisted hypercoagulability after surgery for benign diseases: a prospective cohort study

**DOI:** 10.1186/s12893-016-0128-3

**Published:** 2016-04-06

**Authors:** Jan Ulrych, Tomas Kvasnicka, Vladimir Fryba, Martin Komarc, Ivana Malikova, Filip Burget, Radka Brzezkova, Jan Kvasnicka, Zdenek Krska, Jan Kvasnicka

**Affiliations:** 1st Department of Surgery - Department of Abdominal, Thoracic Surgery and Traumatology; First Faculty of Medicine, Charles University in Prague and General University Hospital in Prague, Prague, Czech Republic; Thrombotic Center, Institute of Medical Biochemistry and Laboratory Diagnostics, General University Hospital, Charles University, Prague, Czech Republic; Institute of Biophysics and Informatics, First Faculty of Medicine, Charles University in Prague, Prague, Czech Republic; Department of Methodology, Faculty of Physical Education and Sport, Charles University in Prague, Prague, Czech Republic; Thrombotic Centre of Institute of Medical Biochemistry and Laboratory Diagnostics, General University Hospital, Karlovo namesti 32, 121 11 Prague, Prague 2 Czech Republic

**Keywords:** Hernia repair, Laparoscopic cholecystectomy, Acute phase reaction, Hypercoagulability, Venous thromboembolism (VTE)

## Abstract

**Background:**

Surgery for benign disease is associated with a low-risk of developing venous thromboembolism (VTE). Despite a relatively low incidence of postoperative VTE in patients after elective cholecystectomy and abdominal hernia repair there are data proving hypercoagulability in the early postoperative period. We focused on assessment of the systemic inflammatory response and coagulation status in these surgical patients after hospital discharge.

**Methods:**

Prospectively, patients who underwent surgery for benign disease were included. Two hundred sixteen patients were enrolled - 90 patients in laparoscopic cholecystectomy (LC) group and 126 patients in hernia surgery (HS) group. Risk assessment of VTE according to the Caprini risk assessment model was performed in all patients. Prevalence of VTE in postoperative period was observed. Markers of systemic inflammatory response (IL-6, CRP, α-1-acid glycoprotein, transferrin) and coagulation markers (PLT, fibrinogen, prothrombin fragment F1 + 2 and D-dimer) were measured before surgery, on 7–10th postoperative day and on 28–30th postoperative day.

**Results:**

Clinically apparent deep vein thrombosis was diagnosed in only one patient - 0.46 %. Statistically significant elevation of inflammatory markers IL-6, CRP and α-1-acid glycoprotein (*p* < 0.001; all) were proved in both groups of patients on 7–10th postoperative day. Statistically significant elevation of coagulation markers PLT, fibrinogen, prothrombin fragment F1 + 2 and D-dimer (*p* < 0.001; all) were proved in LC and HS groups on 7–10th postoperative day. No statistical difference was observed in IL-6, CRP and α-1-acid glycoprotein levels a month after surgery as compared with preoperative levels within each group. Statistically significant elevation of fibrinogen and prothrombin fragment F1 + 2 levels (*p* < 0.001; both) persisted on 28–30th postoperative day in both groups. Persisted elevation of D-dimer levels was proved only in HS group (*p* < 0.001), not in LC group (*p* = 0.138), a month after surgery.

**Conclusions:**

Activated systemic inflammatory response and hypercoagulable condition were verified in patients after laparoscopic cholecystectomy and hernia surgery after their hospital discharge. Hypercoagulability persisted even a month after surgery. Nevertheless, we observed very low prevalence of clinically apparent VTE in patients with in-hospital postoperative VTE prophylaxis.

**Trial registration:**

Trials of the Czech Ministry of Health No. RVO-VFN64165 and NT 13251-4.

## Background

Abdominal hernia repair, cholecystectomy and varicose vein operation have been considered as low-risk surgery for developing venous thromboembolism (VTE). In the literature, the reported incidence of VTE ranges from 0.08 %–1.2 % in patients undergoing hernia repair, 0.28 %–0.53 % in patients after elective laparoscopic cholecystectomy and 0.18 % in patients after varicose vein operation [1–4]. The risk of VTE is usually determined according to the Caprini risk assessment model. Routine pharmacologic prophylaxis of VTE in general surgery is recommended only for moderate and high risk patients [5]. In patients with very low and low risk of VTE only, early mobilisation and/or mechanical prophylaxis of VTE (elastic compression stockings) is recommended [5]. There is no recommendation in the 9th ACCP guidelines regarding the duration of VTE prophylaxis in surgery for benign diseases and no definite consensus exists because of the absence of sufficient numbers of valid data. Postoperative prophylaxis in duration of 7–10 days or VTE prophylaxis until full mobilisation of the patient is recommended most frequently in low risk surgery [6]. Some authors demonstrate the feasibility of short postoperative VTE prophylaxis without higher rates of venous thromboembolism. They recommend individualised duration of VTE prophylaxis administration based on stratification of the VTE risk in concrete patients [7, 8]. Despite relatively low incidences of postoperative VTE in patients after elective cholecystectomy and abdominal hernia repair, there are data proving hypercoagulability in the early postoperative period. Hypercoagulable status in patients after elective laparoscopic and open surgery has been well-documented in numerous publications [9–11]. However, most of the previous studies have monitored coagulation status and systemic inflammatory response only in the early postoperative period, most often within 72 h after surgery [9–17]. We focused on assessment of the systemic inflammatory response and coagulation status in patients after open hernia repair and after laparoscopic cholecystectomy one week and one month after surgery, when these patients were already predominantly out-of-hospital. The patients were compared with the grade of thromboembolic risk according to a new adaptation of the Caprini risk assessment model [5].

## Methods

### Patients

The study was performed as a prospective nonrandomized study from January 2011 to April 2012 and the patients undergoing surgery for benign diseases were included. Altogether 232 consecutive patients were enrolled in our study. 16 patients were dropped out because of incomplete laboratory tests. Thus, we assessed 216 patients, of whom 90 patients underwent a laparoscopic cholecystectomy (LC) and 126 patients underwent an elective abdominal hernia surgery (HS). Inclusion criteria: elective laparoscopic surgery for symptomatic gall stone disease or elective open surgery for abdominal hernia. Exclusion criteria: acute cholecystitis, incarcerated hernia, age < 18 or > 90 years, malignancy, VTE in previous 6 months. Basic clinical parameters – age, height, weight, body mass index (BMI), blood pressure, pulse – were noted in every patient. A new adaptation of the Caprini risk assessment model was used to assess the VTE risk in every patient preoperatively [5]. Each patient was classified into one of four risk groups – very low risk, low risk, moderate risk and high risk. VTE prophylaxis comprised the use of lower extremity bandages and administration of low-molecular-weight heparin (LMWH) subcutaneously. LMWH was administered once a day up to the hospital discharge or full mobilisation of the patient. In the postoperative period, clinical parameters were observed – length of hospital stay, duration of LMWH administration, postoperative complications and clinically apparent VTE in 30 days’ postoperative period. Postoperative complications were assessed according to Clavien-Dindo classification [18]. Clinical assessment of deep vein thrombosis risk was performed according to the Wells score system [19]. Patients with a Wells score of > 2 warrant ultrasound examination of legs to verify deep vein thrombosis. Genetic examination was carried out in all patients to exclude hereditary thrombophilia – Factor V Leiden mutation (FVL) and factor II – prothrombin 20210G > A mutation. For monitoring of systemic inflammatory response, these markers were used: interleukin 6 (IL-6), C-reactive protein (CRP), α-1-acid glycoprotein (Oroso), transferrin (Trf). For monitoring of postoperative hypercoagulability, these markers were used: platelets count (PLT), fibrinogen (Fbg), prothrombin fragment F1 + 2 (F1 + 2) and D-dimer. Markers of systemic inflammatory response and coagulation markers were measured preoperatively, on 7-10th postoperative day and on 28–30th postoperative day. The study was approved by the ethics committee of the 1st Faculty of Medicine of Charles University and General University Hospital in Prague. Each participant gave written informed consent to be included in the study and with the anonymous publication of data.

### Laboratory tests

Venous blood samples were obtained and administered to Vacutainer™ pneumatic tubes (Becton Dickinson, Meylan Cedex, France). EDTA blood was used for full blood count. Hemogram was determined with Beckman Coulter UniCel®DxH800 Cellular Analysis System (Beckman Coulter, Miami, Florida, USA). Citrated platelet poor plasma was prepared by double centrifugation at 2500 × g for 15 min at room temperature and stored in 1.0 mL aliquots at − 80 °C until testing fibrinogen, prothrombin fragment F1 + F2 and D-dimer. Coagulation tests were carried out on a Behring coagulation system (BCS) ™ analyzer (Dade Behring, Marburg, Germany). Determination of F IIa (trombin) inhibition and concentration level of prothrombin fragment F1 + 2 were carried out according to the manufacturer‘s instructions using the MRX Microplate Reader™ microphotometer (Dynatech Laboratories, Chantilly, USA). Fibrinogen was determined using the Fibrinogen reagent kit (product supplied By Technoclone, Austria). Prothrombin fragment F1 + 2 was determined using the Enzygnost® F 1 + 2 micro kit (product supplied by Dade Behring, Marburg, Germany). D-dimer was determined using the ELISA D dimer kit (product supplied by Boehring-Mannheim, Germany). Acute phase proteins (CRP, Oroso, Trf) were determined using the Nephelometer BNII (Dade Behring, Marburg, Germany).

### Genetic tests

Genome DNA was extracted from leukocytes in peripheral blood and isolated using the MagNA Pure LC Nucleic Acid Extraction system™ with a MagNA Pure DNA Isolation Kit I™. DNA was isolated according to the MagNA Pure High-Performance DNA Extraction™ protocol (all products supplied by Roche Diagnostics, Mannheim, Germany). Monitored mutations were determined using PCR in a process called FRET (Fluorescence Resonance Energy Transfer). Tests were performed using the LightCycler® 480 System with LC® 480 Genotyping Master kits (all products supplied by Roche Diagnostics, Mannheim, Germany). Specific primers and fluorescently labelled probes were designed in cooperation with TIB MOLBIOL (Berlin, Germany), where they were custom made.

### Statistical analysis

Basic descriptive statistics within two groups of patients (cholecystectomy, hernia repair) were computed for all variables, which were subsequently tested for normality using Kolmogorov-Smirnov and Shapiro-Wilk tests. Changes in the non-normally distributed systemic inflammatory response markers (IL6, CRP, Oroso, Trf) and coagulant markers (Fbg, D-dimer, PLT, F1 + F2) at three different time points (preoperative, 1 week after surgery and 4 weeks after surgery) were assessed separately within each group of patients by the Friedmann test, followed by a series of Wilcoxon signed rank tests with Bonferroni correction as a post-hoc comparison. *P*-values below 0.05 were considered to be statistically significant. Statistical analysis was performed using SPSS version 22 (SPSS Inc., Chicago, IL, USA).

## Results

### Basic characteristics of our study group

Basic characteristics of our study group are depicted in Table 1. Only patients with uncomplicated symptomatic gallstone disease were included in LC group. All cholecystectomies were finished laparoscopically. The hernia surgery group comprises patients with different types of abdominal hernias – inguinal hernia *n* = 65 (51.59 %), incisional hernia *n* = 26 (20.63 %), umbilical hernia *n* = 17 (13.49 %), epigastric hernia *n* = 17 (13.49 %), femoral hernia *n* = 1 (0.79 %). Hernia repair was performed either as a conventional open hernioplasty n = 95 (75.40 %) or open hernia repair with mesh *n* = 31 (24.60 %). Risk assessment of VTE in the study population, as per the Caprini risk assessment model, is shown in Table 2.

**Table 1:** Basic characteristics of study population

	Cholecystectomy	Hernia surgery
	Median (IQR)	Median (IQR)
Age (years)	53.00 (38.00 – 61.25)	58.00 (41.50 – 66.00)
Height (cm)	168.50 (164.75 – 176.50)	175.00 (170.00 – 180.00)
Weight (kg)	75.00 (63.00 – 85.00)	84.00 (74.00 – 92.50)
BMI (kg/m^2^)	26.15 (22.75 – 28.45)	27.00 (24.75 – 29.90)
BP syst. (mmHg)	120.00 (120.00 – 130.00)	120.00 (120.00 – 135.00)
BP diast. (mmHg)	80.00 (78.75 – 80.00)	80.00 (75.00 – 80.00)
Puls (/min.)	75.00 (69.50 – 76.00)	76.00 (70.00 – 76.00)

Notes: *IQR* interquartile range; *BMI* body mass index; *BP* blood pressure

**Table 2:** Classification of VTE risk in study population according to the Caprini risk assessment model

Patient stratification	Cholecystectomy no. of pts. (%)	Hernia surgery no. of pts. (%)
Very low risk (0 point)	0	0
Low risk (1–2 points)	8 (8.9 %)	17 (13.5 %)
Moderate risk (3–4 points)	39 (43.3 %)	60 (47.6 %)
High risk (>5 points)	43 (47.8 %)	49 (38.9 %)

### Postoperative course

The median length of postoperative hospital stay for both groups was 3 days. Equally, the median length of LMWH administration for patients was 3 days after both types of surgery. Postoperative complications occurred in 6 patients (2.78 %) - wound hematoma *n* = 4 (1.85 %), postoperative bronchopneumonia *n* = 1 (0.46 %), clinically apparent deep vein thrombosis *n* = 1 (0.46 %). Classification of postoperative complications is shown in Table 3.

**Table 3:** Postoperative course and postoperative complications

	Cholecystectomy	Hernia surgery
Postoperative complications according to Clavien-Dindo classification (No. of pts.)		
Clavien-Dindo II	Postoperative pneumonia (*n* = 1)	Wound hematoma (*n* = 2) DVT (*n* = 1)
Clavien-Dindo IIIa		Wound hematoma (*n* = 1)
Clavien-Dindo IIIb		Wound hematoma (*n* = 1)
Length of hospital stay, days median (range)	3 (2–9)	3 (1–10)
Length of LMWH administration, days median (range)	3 (1–9)	3 (1–10)

Notes: *DVT* deep vein thrombosis; *LMWH* low molecular weight heparin

### Hereditary trombophilia

Hereditary thrombophilia was detected in 18 patients (8.33 %) – FVL mutation (heterozygote) in 11 patients (5.12 %), Factor II 20210G > A mutation (heterozygote) in 6 patients (2.80 %) and, in one patient, both mutations (FVL and F II 20210G > A heterozygote) were proved. The hereditary thrombophilic status was diagnosed in 8 patients (8.89 %) in LC group and in 10 patients (7.94 %) in HS group.

### Acute phase proteins and coagulation markers

#### Hernia surgery group

On postoperative day 7–10, statistically significant elevation of most acute phase proteins were proved as compared to preoperative levels - IL-6 (*p* < 0.001), CRP (*p* < 0.001) and α-1-acid glycoprotein (*p* < 0.001). Transferrin levels did not differ significantly (p = 0.074). On day 28–30 after surgery, no significant difference in levels of IL-6 (*p* = 0.281), CRP (*p* = 0.267) and α-1-acid glycoprotein (*p* = 0.527) were observed as compared with preoperative levels; however, transferrin significantly decreased compared to the preoperative levels (*p* = 0.001). A statistically significant increase in coagulation marker levels was proved on day 7–10 after surgery – PLT, fibrinogen, prothrombin fragment F1 + 2 and D-dimer (*p* < 0.001; all). On postoperative day 28-30, only PLT levels did not differ significantly compared to preoperative PLT levels (*p* = 0.088). Persisted elevation of fibrinogen, prothrombin fragment F1 + 2 and D-dimer levels (*p* < 0.001, all) were observed even one month after hernia surgery (Table 4).

**Table 4:** Results of monitoring markers

		Cholecystectomy		Hernia surgery	
		Median (IQR)	*p*-value	Median (IQR)	*p*-value
Fbg (g/l)	Fbg^*^	3.56 (3.17–3.90)		3.45 (3.05–3.95)	
	Fbg^†^	4.16 (3.66–4.66)	p_*†_ < 0.001	4.37 (3.71–5.09)	p_*†_ < 0.001
	Fbg^‡^	3.63 (3.25–4.01)	p_*‡_ = 0.002	3.58 (3.20–4.22)	p_*‡_ < 0.001
D-dimer (μg/l)	D-dimer^*^	96.50 (57.00–139.25)		104.00 (62.00–161.00)	
	D-dimer^†^	409.00 (273.50–577.00)	p_*†_ < 0.001	318.50 (173.75–554.25)	p_*†_ < 0.001
	D-dimer^‡^	109.50 (67.00–155.50)	p_*‡_ = 0.138	123.00 (71.50–216.00)	p_*‡_ < 0.001
PLT (10^9^/l)	PLT^*^	230.00 (200.50–259.50)		212.00 (181.50–248.00)	
	PLT^†^	267.00 (234.50–303.50)	p_*†_ < 0.001	262.00 (225.00–312.00)	p_*†_ < 0.001
	PLT^‡^	227.00 (206.00–275.00)	p_*‡_ = 0.042	216.00 (183.25–267.00)	p_*‡_ = 0.088
F1 + F2 (nmol/l)	F1 + 2^*^	0.17 (0.14–0.21)		0.18 (0.14–0.24)	
	F1 + 2^†^	0.24 (0.19–0.30)	p_*†_ < 0.001	0.26 (0.19–0.30)	p_*†_ < 0.001
	F1 + 2^‡^	0.21 (0.18–0.25)	p_*‡_ = 0.002	0.23 (0.19–0.31)	p_*‡_ < 0.001
Trf (g/l)	Trf^*^	2.33 (2.08–2.65)		2.29 (2.00–2.55)	
	Trf^†^	2.42 (2.28–2.72)	p_*†_ = 0.057	2.36 (2.10–2.74)	p_*†_ = 0.074
	Trf^‡^	2.09 (1.89–2.42)	p_*‡_ = 0.007	2.09 (1.96–2.35)	p_*‡_ = 0.001
Orso (g/l)	Oroso^*^	0.71 (0.62–0.81)		0.73 (0.60–0.88)	
	Oroso^†^	0.95 (0.82–1.07)	p_*†_ < 0.001	1.07 (0.85–1.18)	p_*†_ < 0.001
	Oroso^‡^	0.69 (0.63–0.85)	p_*‡_ = 0.201	0.73 (0.57–0.86)	p_*‡_ = 0.527
CRP (mg/l)	CRP^*^	0.90 (0.45–2.10)		1.10 (0.44–2.60)	
	CRP^†^	3.47 (1.90–5.49)	p_*†_ < 0.001	3.85 (1.50–8.13)	p_*†_ < 0.001
	CRP^‡^	0.66 (0.33–1.57)	p_*‡_ = 0.074	1.03 (0.57–2.01)	p_*‡_ = 0.267
IL6 (pg/ml)	IL6^*^	1.35 (0.98–2.06)		1.61 (1.17–2.91)	
	IL6^†^	2.48 (1.71–3.12)	p_*†_ = 0.002	3.30 (1.97–5.40)	p_*†_ < 0.001
	IL6^‡^	1.44 (0.97–1.93)	p_*‡_ = 0.865	2.14 (1.23–2.82)	p_*‡_ = 0.281

Notes: IQR – interquartile range; *p*-values – differences tested by Wilcoxon test; ^*^before surgery; ^†^7–10 days after surgery; ^‡^28–30 days after surgery; p_*†_ – *p*-value of the difference between measurement before surgery and 7–10 days after surgery; p_*‡_ – *p*-value of the difference between measurement before surgery and 28–30 days after surgery

#### Laparoscopic cholecystectomy group

On postoperative day 7–10, statistically significant elevation of acute phase proteins were proved as compared to preoperative levels - IL-6 (*p* = 0.002), CRP (*p* < 0.001) and α-1-acid glycoprotein (*p* < 0.001). Transferrin levels did not differ significantly (p = 0.057). On day 28–30 after surgery, no significant difference in levels of IL-6 (*p* = 0.865), CRP (*p* = 0.074) and α-1-acid glycoprotein (*p* = 0.201) were observed as compared with preoperative levels; however, transferrin significantly decreased compared to the preoperative levels (*p* = 0.007). A statistically significant increase in coagulation marker levels was proved on day 7–10 after surgery – PLT, fibrinogen, prothrombin fragment F1 + 2 and D-dimer (*p* < 0.001; all). On postoperative day 28–30, PLT levels (*p* = 0.042) and D-dimer levels (*p* = 0.138) did not differ significantly compared to preoperative levels. However, persisted elevation of fibrinogen, prothrombin fragment F1 + 2 levels (*p* = 0.002; both) were observed even one month after laparoscopic cholecystectomy (Table 4).

## Discussion

Given the absence of data related to the duration of postoperative systemic inflammatory response and postoperative hypercoagulability in patients after surgery for benign disease, we focused on the monitoring of inflammatory markers and coagulation markers in patients after open hernia surgery (HS) and after laparoscopic cholecystectomy (LC). Basic characteristics of the study population were assessed separately for more detailed information, not for comparison. Most patients in the HS group and LC group were classified as moderate risk (47.6 % and 43.3 %, respectively) and high risk (38.9 % and 47.8 %, respectively) according to the new adaptation of the Caprini risk assessment model. The postoperative risk of VTE can also be increased by hereditary thrombophilia and postoperative complications. FVL and Factor II 20210G > A belong among the most frequent hereditary thrombophilia predispositions with a moderate risk of VTE. The frequency of these two hereditary thrombophilic conditions in our study population corresponds to their prevalence in the Czech population [20]. Postoperative complications are an important factor which can affect postoperative systemic inflammatory response and prolong postoperative hypercoagulability. The postoperative complications were acceptably low in our study group. Just et al. reported a complication rate of 2.7 % after hernia surgery and Ahmad et al. reported a complication rate of 5.4 % after laparoscopic cholecystectomy [21, 22]. The median length of LMWH administration is identical to the median length of hospital stay (3 days). This fact corresponds with the generally performed clinical practice of administering pharmacologic VTE prophylaxis only in hospital. There is no definite recommendation concerning the length of administration of pharmacologic VTE prophylaxis in patients operated on for benign diseases. Tincani et al. published a study in which they observed the prevalence of postoperative VTE in patients undergoing laparoscopic surgery for a benign disease and compared the two different pharmacologic thromboprophylaxis regimens; a short-term pharmacologic thromboprophylaxis (3–4 days in hospital) or an extended-duration pharmacologic thromboprophylaxis (in hospital + 7 days after discharge). The prevalence of deep vein thrombosis in patients with the short-term VTE prophylaxis was 0.95 % and the authors concluded that the extended-duration VTE prophylaxis did not provide any benefit [7]. Our results (prevalence of postoperative VTE in our study population – 0.46 %) support the conclusion that in patients undergoing an uncomplicated surgery for a benign disease, the risk of clinically apparent VTE after hospital discharge is very low (in the case of correct VTE prophylaxis in hospital).

Surgery is associated with the development of a stress reaction by the organism which is called systemic inflammatory response or acute phase reaction. Systemic inflammatory response is a common reaction of the organism to any trauma. Nevertheless, the course and the intensity of the systemic inflammatory response can be influenced by many other factors. Age, obesity, comorbidities and smoking are preoperative factors stimulating the systemic inflammatory response [23]. Systemic inflammatory response markers can be influenced by postoperative complications yet, on the contrary, no impact of the anaesthesia type on the postoperative CRP levels was proved [24, 25]. Cortisol, IL-6, white blood cell count (WCC) and CRP belong to the most frequently studied systemic inflammatory response markers. Only IL-6 and CRP, however, allow for differentiating the relevance and the intensity of the systemic inflammatory response caused by surgical trauma [26]. Several authors have already published results of the systemic inflammatory response in patients after abdominal hernia surgery in the early postoperative period (within 48 h after surgery) [12–14, 27–30]. Only Akhtar et al. studied the systemic inflammatory response in their patients after laparoscopic and open inguinal hernia repair up to the 5th postoperative day [31]. Our results support the hypothesis that activated systemic inflammatory response persists after open hernia surgery even on postoperative day 7–10. Levels of inflammatory markers (IL-6, CRP, α-1-acid glycoprotein) were statistically significantly elevated on postoperative day 7–10 as compared to preoperative levels. However, a month after the surgery, the inflammatory markers returned to the preoperative levels, which reflect the subsided systemic inflammatory response. Regarding cholecystectomy, laparoscopic surgery is associated with lower systemic inflammatory response compared to open cholecystectomy [15–17]. More significant systemic inflammatory responses in patients after open cholecystectomy are related to the magnitude of tissue trauma as a result of laparotomy. Di Vita et al. and Sista et al. measured the systemic inflammatory response after open and laparoscopic cholecystectomy till 7th and 12th postoperative day, respectively. These authors noted that IL-6 and CRP levels normalize by about the 6th or 7th postoperative day [15, 32]. On the contrary, our results demonstrate persisted systemic inflammatory response after laparoscopic cholecystectomy on postoperative day 7–10. Significantly elevated inflammatory markers (IL-6, CRP, α-1-acid glycoprotein) were proven at that time. A month after the surgery, the systemic inflammatory response had subsided and the inflammatory markers dropped to baseline levels.

Postoperative hypercoagulability is an intrinsic response to surgical trauma. In general, surgical trauma induces a secondary postoperative thrombocytosis, elevated levels of fibrinogen, prothrombin fragment F1 + 2 and D-dimer [33–35]. Postoperative hypercoagulability and levels of coagulation markers can be modified by the systemic inflammatory response, type of surgery, postoperative complications and postoperative drug administration. Dedej et al. described in patients after open surgery a decrease in PLT levels 72 h postoperatively [10]. PLT count decrease in the early postoperative period has been explained by postoperative platelet consumption and by postoperative VTE prophylaxis – administration of unfractionated heparin or LMWH [10]. Fibrinogen is a marker of activated coagulation and fibrinogen also belongs to acute phase proteins. Prothrombin fragment F1 + 2 corresponds with the formed volume of thrombin and can be regarded as a coagulation activity marker [36]. D-dimer forms during the plasmin-induced breakdown of fibrin in the fibrinolytic pathway. D-dimer is considered a common marker of coagulation activation, particularly a marker of fibrinolysis. Elevated coagulation markers have already been documented in the early postoperative period in patients after cholecystectomy and hernia surgery. Return of plasmatic fibrinogen to the preoperative level was described by Di Vita et al. on postoperative day 7 after laparoscopic and open cholecystectomy as well [15]. Patients after open cholecystectomy have higher plasmatic prothrombin fragment F1 + 2 levels in the early postoperative period compared to patients after laparoscopic cholecystectomy [34, 35]. However, there are no data concerning hypercoagulability duration after surgery for benign diseases. We observed interesting results in both surgery groups of patients. Persisted hypercoagulability was proven in hernia surgery group patients on postoperative day 7–10. Statistically significantly elevated levels of all coagulation markers (PLT count, levels of fibrinogen, prothrombin fragment F1 + 2 and D-dimer) were measured. On postoperative day 28–30, significantly elevated levels of fibrinogen, prothrombin fragment F1 + 2 and D-dimer were persistent in patients after hernia surgery. Postoperative hypercoagulability persisted in cholecystectomy group patients as well. All coagulation markers were statistically significantly elevated compared to preoperative baseline levels on postoperative day 7–10. A month after the laparoscopic cholecystectomy decrease and normalization of PLT count and D-dimer levels were observed. However, persisted markers of activated coagulation (fibrinogen, prothrombin fragment F1 + 2) were proven in cholecystectomy group patients on 28th–30th postoperative day. Prolonged elevation of D-dimer levels and persisted activation of fibrinolysis in patients after open hernia repair can be explained by more extensive soft tissue trauma and the implantation of a foreign body – mesh. The hypercoagulability status was proven in our patients not only a week after but also, surprisingly, a month after hernia surgery and laparoscopic cholecystectomy.

## Conclusion

Despite the long persisting hypercoagulability in patients after laparoscopic cholecystectomy and hernia surgery, the prevalence of clinically apparent VTE was low in our study population – 0.46 %. Our data support short-term (in hospital) postoperative VTE prophylaxis in moderate and high risk patients undergoing hernia repair or cholecystectomy.

## Abbreviations

ACCP: American College of Chest Physicians
BMI: body mass index
BP: blood pressure
CRP: C reactive protein
Fbg: fibrinogen
FVL: Factor V Leiden mutation
F1 + 2: prothrombin fragment F1 + 2
HS: hernia surgery
IL-1: interleukin 1
IL-6: interleukin 6
IQR: interquartile range
LC: laparoscopic cholecystectomy
LMWH: low molecular weight heparin
Oroso: α-1-acid glycoprotein
PLT: platelets
SD: standard deviation
TNFα: tumor necrosis factor α
Trf: transferrin
VTE: venous thromboembolism
